# Crystal structure of 2-(2-amino­phen­yl)-1,3-benzoxazole

**DOI:** 10.1107/S2056989015000481

**Published:** 2015-01-21

**Authors:** Imelda Pérez-Pérez, Diego Martínez-Otero, Susana Rojas-Lima, Heraclio López-Ruiz

**Affiliations:** a Área Académica de Química, Universidad Autónoma del Estado de Hidalgo, km. 4.5 Carretera Pachuca-Tulancingo, Mineral de la Reforma, Hidalgo CP 42184, Mexico; b Centro Conjunto de Investigación en Química Sustentable, UAEM-UNAM, carretera Toluca-Atlacomulco km. 14.5, CP 50200, Toluca, Estado de México, Mexico

**Keywords:** crystal structure, benzoxazole, N—H⋯N hydrogen bonding

## Abstract

Crystals of the title compound, C_13_H_10_N_2_O, were grown from a di­chloro­methane/ketone/methanol solvent mixture. It crystallizes with two mol­ecules, *A* and *B*, in the asymmetric unit with very similar almost planar conformations [dihedral angles between the ring planes = 0.74 (8) and 0.67 (6)° for mol­ecules *A* and *B*, respectively; r.m.s. overlay fit = 0.019 Å]. Each mol­ecule features an intra­molecular N—H⋯N hydrogen bond, which closes an *S*(6) ring and therefore establishes a *syn* relationship for the N atoms. In the crystal, mol­ecules are linked by N—H⋯N hydrogen bonds, generating [100] chains containing alternating *A* and *B* mol­ecules. Weak aromatic π–π stacking [minimum centroid–centroid separation = 3.6212 (9) Å] links the chains into a three-dimensional network.

## Abstract 2

The two mol­ecules of 2-(2-amino­phen­yl)-1,3-benzoxazole in the asymmetric unit feature an intra­molecular N—H⋯N hydrogen bond, which closes an *S*(6) ring and therefore establishes a *syn* relationship for the N atoms. In the crystal, mol­ecules are linked by N—H⋯N hydrogen bonds, generating [100] chains containing alternating *A* and *B* mol­ecules.

## Chemical context   

Benzimidazole, benzoxazole, and benzo­thia­zole derivatives are key components in many bioactive compounds of both natural and synthetic origin; many are active components of biocides such as bactericides, fungicides, insecticides and anti­carcinogens (Kumar-Samota & Seth, 2010 ▸). Benzoxazole derivatives have been used as building blocks for biochemical and pharmaceutical agents, as well as dyes, fluorescent brightening agents, biomarkers and biosensors (Costa *et al.* 2007 ▸ and Tong *et al.* 2005 ▸). 
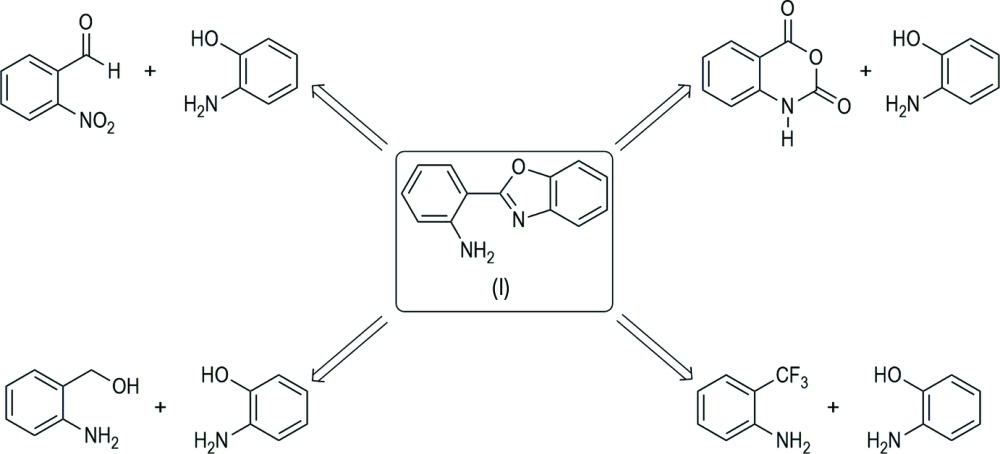


In this context, 2-(2-amino­phen­yl)benzoxazole has shown considerable growth inhibition with respect to fungi and gram-positive and gram-negative bacteria (Elnima *et al.* 1981 ▸). For this reason, several methods have been described for the synthesis of these heterocyclic compounds, some of which are summarized in the Scheme, which shows the retrosynthesis for the preparation of the title compound, (I). For example, Gajare *et al.* (2000 ▸) described a procedure for the preparation of 2-(*o*-amino­phen­yl)oxazolines from isatoic anhydride and 2-amino­alcohols at reflux of PhCl mediated *via* a natural kaolinitic clay catalyst; a slightly modified procedure has been describe by Button & Gossage (2003 ▸) using zinc chloride as a catalyst. Qiao *et al.* (2011 ▸) described the synthesis of benzoxazole *via* the reaction of anionically activated tri­fluoro­methyl groups with amino nucleophiles under mild aqueous conditions. Recently, Khalafi-Nezhad & Panahi (2014 ▸) reported an efficient approach for the preparation of benzoxazole derivatives, *via* acceptorless de­hydrogenative coupling of alcohols with 2-amino­phenol using an Ru catalytic system.

In the present work, as part of our ongoing studies of heterocyclic compounds (López-Ruiz *et al.*, 2011 ▸, 2013 ▸; de la Cerda-Pedro *et al.*, 2014 ▸), we report the synthesis of 2-(2-amino­phen­yl)benzoxazole, we analyse its mol­ecular structure, as well as its weak inter­molecular inter­actions in mol­ecular packing, which could be useful in the understanding of their mode of action in pharmaceutical science, as well as in the design of materials with specific functions. The title compound has been previously reported by Button & Gossage (2003 ▸) from isatoic anhydride and 2-aminophenol but its crystal structure has not been described.

## Structural commentary   

**Figure 1:**
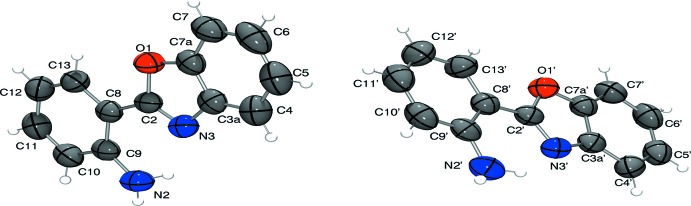
The asymmetric unit of (I) with displacement ellipsoids drawn at the 50% probability level (left: mol­ecule *A* and right: mol­ecule *B*)

Compound (I) crystallized in the monoclinic space group *P*2_1_/*c* with two independent mol­ecules (*A* and *B*) in the asymmetric unit (Fig. 1 ▸). The orientation of the amino group can be described using as a basis the carbon atom C9, this orientation is *syn* to the nitro­gen atom N3 and *anti* for the oxygen atom O1.

The skeleton of each mol­ecule is practically planar: to analyse the planarity of the mol­ecule we use the torsion angle N3—C2—C8—C9, indicating the rotation of the aromatic ring C8—C13: these angles are −1.2 (2) and 0.9 (2)° for mol­ecules *A* and *B*, respectively. The dihedral angles between the benzene ring and the fused ring system are 0.74 (8) and 0.67 (6)° for mol­ecules *A* and *B*, respectively. The two independent mol­ecules are very similar, with an r.m.s. overlay fit of 0.019 Å.

## Supra­molecular features   

**Figure 2:**
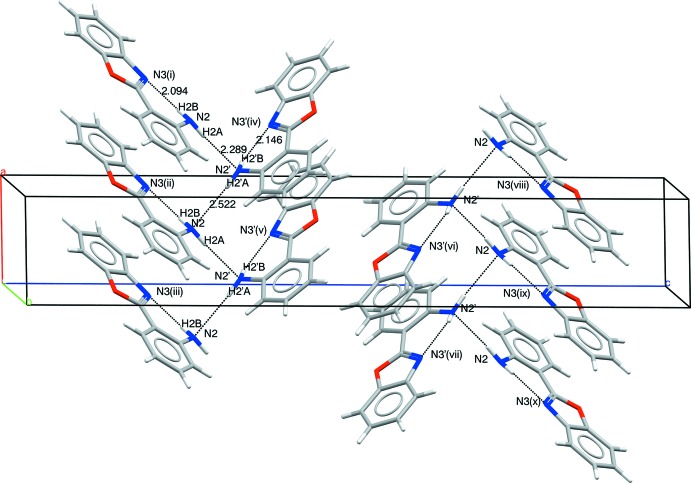
Crystal packing for (I), showing the formation of [100] chains. [Symmetry codes: (i) 2 − *x*, −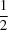 + *y*, 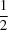 − *z*; (ii) 1 − *x*, −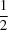 + *y*, 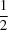 − *z*; (iii) −*x*, −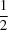 + *y*, 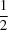 − *z*; (iv) 1 + *x*, *y*, *z*; (v) *x*, *y*, *z*; (vi) 1 − *x*, 1 − *y*, 1 − *z*; (vii) −*x*, 1 − *y*, 1 − *z*; (viii) 1 + *x*, 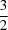 − *y*, 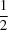 + *z*; (ix) *x*, 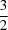 − *y*, 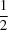 + *z*; (x) −1 + *x*, 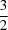 − *y*, 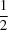 + *z*.]

In the crystal, each NH_2_ group forms an intra­molecular hydrogen bond of the type N2—H2*B*⋯N3 (Table 1 ▸) with an H⋯N distance of 2.094 (18) Å in mol­ecule *A* and 2.146 (18) Å in mol­ecule *B*, and an inter­molecular N2—H2*A*⋯N2 hydrogen bond with a distance of 2.289 (15) Å for N2—H2*A*⋯N2′ and 2.522 (16) Å for N2′—H2*A*′⋯N2, forming zigzag chains propagating in the [100] direction and containing alternating *A* and *B* mol­ecules (Fig. 2 ▸). Weak aromatic π–π stacking [minimum centroid–centroid separation = 3.6212 (9) Å] links the chains into a three-dimensional network.

## Synthesis and crystallization   

500 mg (3.00 mmol) of isatoic anhydride were dissolved in 50 mL of *m*-xylene then 390 mg (3.60 mmol) of *o*-amino­phenol were added followed by the addition of 0.30 ml (10% mol) of a solution of ZnCl_2_ (1 *M*). The mixture was then stirred and heated slowly to reflux temperature during 18 h. The crude reaction product was concentrated on a rotary evaporator with an azeotropic mixture of AcOEt/xylene to obtain a reddish brown solid which was dissolved in EtOAc and washed with 10% aq. NaCl solution. The crude reaction product was purified by column chromatography to give 356 mg (55%) of the amine (I) as a white solid m.p. = 381–382 K (literature value 379–381 K; Button & Gossage, 2003 ▸); IR (film) *γ*_max_ cm^−1^: 3408 NH_2_, 3051 C—H(arom), 1624 C=N; (literature value IR: 1620 cm^−1^; Button & Gossage, 2003 ▸); ^1^H NMR (CDCl_3_, 400 MHz): δ = 6.20 (*br s*, 2H, NH_2_), 6.79 (*m*, 2H), 7.29 (*m*, 1H), 7.33 (*m*, 2H), 757 (*m*, 1H), 7.72 (*m*, 1H), 8.09 (*dd*, J = 1.6 Hz, J = 8.2 Hz, 1H); ^13^C NMR (CDCl_3_, 100 MHz) δ = 108.7, 110.4, 116.3, 116.8, 119.4, 124.3, 124.8, 128.8, 132.5, 141.9, 147.9, 149.3, 163.2 [Literature: Button & Gossage (2003 ▸); ^1^H NMR δ = 6.15 (*br s*, 2H, –NH_2_), 6.74 (*m*, 2H, ArH), 7.28 (*m*, 3H, ArH), 7.51 (*m*, 1H, ArH), 7.67 (*m*, 1H, ArH), 8.03 (*m*, 1H, ArH). ^13^C{^1^H} NMR δ = 108.7, 110.3, 116.3, 116.8, 119.4, 124.3, 124.7, 128.8, 132.4, 141.9, 147.9, 149.3, 163.2]. Analysis calculated for C_13_H_10_N_2_O: C, 74.27; H, 4.79%; Found: C, 74.43; H, 5.05%.

The single crystal used in the experiment was obtained by the method of liquid–liquid diffusion by slow evaporation. The pure compound was dissolved in the minimum amount of di­chloro­methane to be added by the walls of the tube the same amount of acetone followed by methanol. The tube was sealed to leave the solution in a vibration-free environment at room temperature. After a few days, the solution had evaporated, leaving colourless blocks of the title compound.

## Refinement   

Crystal data, data collection and structure refinement details are summarized in Table 2 ▸. C-bond H atoms were placed in calculated positions and allowed to ride on their carrier atoms, with C—H = 0.93 Å (aromatic CH) and with *U_iso_*(H) = 1.2*U_eq_*(C). Hydrogen atoms of the amine group were found in a difference map and refined freely.

## Supplementary Material

Crystal structure: contains datablock(s) I. DOI: 10.1107/S2056989015000481/hb7320sup1.cif

Structure factors: contains datablock(s) I. DOI: 10.1107/S2056989015000481/hb7320Isup2.hkl

Supporting information file. DOI: 10.1107/S2056989015000481/hb7320Isup3.cml

CCDC reference: 1042858

Additional supporting information:  crystallographic information; 3D view; checkCIF report

## Figures and Tables

**Table 1:** Hydrogen-bond geometry (, )

*D*H*A*	*D*H	H*A*	*D* *A*	*D*H*A*
N2H2*A*N2^i^	0.92(2)	2.29(2)	3.202(2)	175(2)
N2H2*B*N3	0.92(1)	2.09(2)	2.7679(19)	129(2)
N2H2*A*N2^ii^	0.86(2)	2.52(2)	3.359(2)	164(2)
N2H2*B*N3	0.89(1)	2.15(2)	2.7913(19)	129(2)

Symmetry codes: (i) 
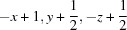
; (ii) 
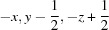
.

**Table 2:** Experimental details

Crystal data	
Chemical formula	C_13_H_10_N_2_O
*M*_r_	210.23
Crystal system, space group	Monoclinic, *P*2_1_/*c*
Temperature (K)	293
*a*, *b*, *c* ()	4.81703(10), 14.8104(3), 29.4801(6)
()	91.3715(18)
*V* (^3^)	2102.57(7)
*Z*	8
Radiation type	Cu *K*
(mm^1^)	0.69
Crystal size (mm)	0.38 0.14 0.11

Data collection	
Diffractometer	Agilent Xcalibur Atlas Gemini
Absorption correction	Analytical [*CrysAlis PRO* (Agilent, 2011 ▸), based on expressions derived by Clark Reid (1995 ▸)]
*T*_min_, *T*_max_	0.742, 0.887
No. of measured, independent and observed [*I* > 2(*I*)] reflections	21894, 4278, 3621
*R*_int_	0.032
(sin /)_max_ (^1^)	0.625

Refinement	
*R*[*F* ^2^ > 2(*F* ^2^)], *wR*(*F* ^2^), *S*	0.043, 0.121, 1.02
No. of reflections	4278
No. of parameters	301
No. of restraints	4
H-atom treatment	H atoms treated by a mixture of independent and constrained refinement
_max_, _min_ (e ^3^)	0.14, 0.16

Computer programs: *CrysAlis PRO* (Agilent, 2011 ▸), *SHELXS97* (Sheldrick, 2008 ▸), *SHELXL2013* (Sheldrick, 2015 ▸) and *OLEX2* (Dolomanov *et al.*, 2009 ▸).

## supplementary crystallographic information

### Crystal data

**Table:**

C_13_H_10_N_2_O	*D*_x_ = 1.328 Mg m^−^^3^
*M**_r_* = 210.23	Melting point: 381 K
Monoclinic, *P*2_1_/*c*	Cu *K*α radiation, λ = 1.54184 Å
*a* = 4.81703 (10) Å	Cell parameters from 7503 reflections
*b* = 14.8104 (3) Å	θ = 3.0–74.3°
*c* = 29.4801 (6) Å	µ = 0.69 mm^−^^1^
β = 91.3715 (18)°	*T* = 293 K
*V* = 2102.57 (7) Å^3^	Block, colourless
*Z* = 8	0.38 × 0.14 × 0.11 mm
*F*(000) = 880	

### Data collection

**Table:**

Agilent Xcalibur Atlas Gemini diffractometer	4278 independent reflections
Radiation source: Enhance (Cu) X-ray Source	3621 reflections with *I* > 2σ(*I*)
Graphite monochromator	*R*_int_ = 0.032
Detector resolution: 10.3659 pixels mm^-1^	θ_max_ = 74.5°, θ_min_ = 3.0°
ω scans	*h* = −6→4
Absorption correction: analytical [*CrysAlis PRO* (Agilent, 2011), based on expressions derived by Clark & Reid (1995)]	*k* = −18→18
*T*_min_ = 0.742, *T*_max_ = 0.887	*l* = −36→36
21894 measured reflections	

### Refinement

**Table:**

Refinement on *F*^2^	4 restraints
Least-squares matrix: full	Hydrogen site location: mixed
*R*[*F*^2^ > 2σ(*F*^2^)] = 0.043	H atoms treated by a mixture of independent and constrained refinement
*wR*(*F*^2^) = 0.121	*w* = 1/[σ^2^(*F*_o_^2^) + (0.0602*P*)^2^ + 0.2656*P*] where *P* = (*F*_o_^2^ + 2*F*_c_^2^)/3
*S* = 1.02	(Δ/σ)_max_ < 0.001
4278 reflections	Δρ_max_ = 0.14 e Å^−^^3^
301 parameters	Δρ_min_ = −0.16 e Å^−^^3^

### Special details

**Table:**

Experimental. Absorption correction: CrysAlisPro, Agilent Technologies, Version 1.171.35.15 (release 03-08-2011 CrysAlis171 .NET) (compiled Aug 3 2011,13:03:54) Analytical numeric absorption correction using a multifaceted crystal model based on expressions derived by R.C. Clark & J.S. Reid. (Clark, R. C. & Reid, J. S. (1995). Acta Cryst. A51, 887-897)
Geometry. All e.s.d.'s (except the e.s.d. in the dihedral angle between two l.s. planes) are estimated using the full covariance matrix. The cell e.s.d.'s are taken into account individually in the estimation of e.s.d.'s in distances, angles and torsion angles; correlations between e.s.d.'s in cell parameters are only used when they are defined by crystal symmetry. An approximate (isotropic) treatment of cell e.s.d.'s is used for estimating e.s.d.'s involving l.s. planes.

### Fractional atomic coordinates and isotropic or equivalent isotropic displacement parameters (Å^2^)

**Table:**

	*x*	*y*	*z*	*U*_iso_*/*U*_eq_	
O1	−0.0170 (2)	0.92286 (7)	0.34568 (3)	0.0625 (3)	
O1'	0.6575 (2)	0.30487 (7)	0.46343 (3)	0.0605 (3)	
N2	0.4198 (3)	0.85716 (10)	0.22601 (4)	0.0678 (3)	
H2A	0.559 (4)	0.8439 (13)	0.2065 (6)	0.081*	
H2B	0.337 (4)	0.8108 (11)	0.2413 (6)	0.081*	
N2'	0.1130 (3)	0.31854 (11)	0.34698 (5)	0.0703 (4)	
H2'A	−0.034 (4)	0.3172 (13)	0.3298 (6)	0.084*	
H2'B	0.180 (4)	0.2642 (11)	0.3545 (7)	0.084*	
N3	0.0403 (2)	0.81678 (8)	0.29206 (4)	0.0575 (3)	
N3'	0.5227 (3)	0.23223 (8)	0.39963 (4)	0.0576 (3)	
C2	0.1126 (3)	0.89555 (9)	0.30691 (4)	0.0532 (3)	
C2'	0.4906 (3)	0.30246 (9)	0.42478 (4)	0.0533 (3)	
C4	−0.3081 (3)	0.70682 (12)	0.32430 (7)	0.0735 (4)	
H4	−0.2870	0.6619	0.3026	0.088*	
C4'	0.8489 (4)	0.09914 (11)	0.41168 (6)	0.0683 (4)	
H4'	0.7961	0.0673	0.3857	0.082*	
C5	−0.4916 (4)	0.69714 (13)	0.35908 (7)	0.0804 (5)	
H5	−0.5976	0.6448	0.3607	0.096*	
C3A	−0.1563 (3)	0.78613 (10)	0.32287 (5)	0.0587 (3)	
C3A'	0.7278 (3)	0.18126 (10)	0.42227 (5)	0.0567 (3)	
C5'	1.0510 (4)	0.06674 (12)	0.44134 (6)	0.0752 (5)	
H5'	1.1357	0.0118	0.4352	0.090*	
C6	−0.5220 (4)	0.76345 (16)	0.39167 (7)	0.0864 (6)	
H6	−0.6470	0.7543	0.4148	0.104*	
C6'	1.1316 (4)	0.11380 (13)	0.48016 (6)	0.0763 (5)	
H6'	1.2695	0.0899	0.4992	0.092*	
C7	−0.3698 (4)	0.84388 (14)	0.39075 (6)	0.0785 (5)	
H7	−0.3887	0.8890	0.4124	0.094*	
C7'	1.0110 (4)	0.19587 (12)	0.49121 (5)	0.0708 (4)	
H7'	1.0631	0.2279	0.5172	0.085*	
C8	0.3115 (3)	0.95868 (9)	0.28832 (4)	0.0536 (3)	
C7A	−0.1899 (3)	0.85106 (11)	0.35532 (5)	0.0607 (3)	
C7A'	0.8096 (3)	0.22647 (10)	0.46119 (5)	0.0574 (3)	
C8'	0.3042 (3)	0.37874 (10)	0.41810 (5)	0.0564 (3)	
C9	0.4607 (3)	0.93665 (10)	0.24915 (4)	0.0553 (3)	
C9'	0.1199 (3)	0.38352 (11)	0.38020 (5)	0.0590 (3)	
C10	0.6493 (3)	1.00084 (11)	0.23331 (5)	0.0670 (4)	
H10	0.7479	0.9883	0.2073	0.080*	
C10'	−0.0505 (4)	0.46002 (13)	0.37630 (6)	0.0734 (4)	
H10'	−0.1725	0.4652	0.3515	0.088*	
C11	0.6923 (4)	1.08145 (12)	0.25509 (6)	0.0734 (4)	
H11	0.8203	1.1223	0.2439	0.088*	
C11'	−0.0415 (4)	0.52712 (13)	0.40806 (7)	0.0805 (5)	
H11'	−0.1578	0.5769	0.4046	0.097*	
C12	0.5471 (4)	1.10261 (11)	0.29352 (6)	0.0722 (4)	
H12	0.5765	1.1575	0.3082	0.087*	
C12'	0.1369 (4)	0.52214 (13)	0.44503 (7)	0.0815 (5)	
H12'	0.1420	0.5681	0.4665	0.098*	
C13	0.3603 (3)	1.04212 (10)	0.30963 (5)	0.0634 (4)	
H13	0.2624	1.0565	0.3355	0.076*	
C13'	0.3072 (4)	0.44845 (11)	0.44974 (6)	0.0709 (4)	
H13'	0.4280	0.4449	0.4748	0.085*	

### Atomic displacement parameters (Å^2^)

**Table:**

	*U*^11^	*U*^22^	*U*^33^	*U*^12^	*U*^13^	*U*^23^
O1	0.0665 (6)	0.0656 (6)	0.0558 (5)	0.0094 (5)	0.0078 (4)	−0.0016 (4)
O1'	0.0618 (6)	0.0648 (6)	0.0549 (5)	0.0045 (5)	−0.0013 (4)	−0.0050 (4)
N2	0.0683 (8)	0.0784 (8)	0.0571 (7)	0.0071 (7)	0.0072 (6)	−0.0083 (6)
N2'	0.0623 (8)	0.0896 (9)	0.0586 (7)	−0.0043 (7)	−0.0046 (6)	0.0023 (7)
N3	0.0519 (7)	0.0619 (6)	0.0587 (6)	0.0081 (5)	−0.0023 (5)	−0.0015 (5)
N3'	0.0572 (7)	0.0621 (6)	0.0535 (6)	−0.0006 (5)	0.0038 (5)	−0.0030 (5)
C2	0.0505 (7)	0.0584 (7)	0.0504 (6)	0.0132 (6)	−0.0028 (5)	0.0011 (5)
C2'	0.0502 (7)	0.0615 (7)	0.0485 (6)	−0.0033 (6)	0.0054 (5)	0.0013 (5)
C4	0.0572 (9)	0.0744 (10)	0.0885 (11)	−0.0002 (7)	−0.0072 (8)	0.0117 (8)
C4'	0.0738 (10)	0.0631 (8)	0.0686 (9)	0.0043 (7)	0.0107 (8)	−0.0016 (7)
C5	0.0558 (9)	0.0857 (11)	0.0994 (13)	−0.0001 (8)	−0.0036 (9)	0.0281 (10)
C3A	0.0468 (7)	0.0663 (8)	0.0627 (8)	0.0096 (6)	−0.0060 (6)	0.0087 (6)
C3A'	0.0555 (8)	0.0593 (7)	0.0558 (7)	−0.0024 (6)	0.0100 (6)	0.0028 (6)
C5'	0.0793 (11)	0.0663 (9)	0.0807 (11)	0.0137 (8)	0.0157 (9)	0.0109 (8)
C6	0.0597 (10)	0.1152 (15)	0.0846 (12)	0.0099 (10)	0.0102 (8)	0.0386 (11)
C6'	0.0704 (10)	0.0836 (11)	0.0749 (10)	0.0141 (8)	0.0036 (8)	0.0226 (9)
C7	0.0724 (11)	0.0958 (12)	0.0676 (9)	0.0158 (9)	0.0120 (8)	0.0119 (9)
C7'	0.0714 (10)	0.0821 (10)	0.0587 (8)	0.0035 (8)	−0.0009 (7)	0.0056 (7)
C8	0.0497 (7)	0.0589 (7)	0.0519 (7)	0.0097 (6)	−0.0045 (5)	0.0056 (5)
C7A	0.0516 (8)	0.0693 (8)	0.0612 (8)	0.0105 (6)	−0.0002 (6)	0.0123 (6)
C7A'	0.0553 (8)	0.0608 (8)	0.0565 (7)	0.0016 (6)	0.0068 (6)	0.0046 (6)
C8'	0.0516 (8)	0.0620 (7)	0.0560 (7)	0.0003 (6)	0.0084 (6)	0.0041 (6)
C9	0.0508 (8)	0.0651 (7)	0.0498 (6)	0.0117 (6)	−0.0053 (5)	0.0033 (6)
C9'	0.0491 (8)	0.0746 (9)	0.0538 (7)	−0.0041 (6)	0.0107 (6)	0.0103 (6)
C10	0.0612 (9)	0.0797 (10)	0.0603 (8)	0.0094 (8)	0.0057 (7)	0.0103 (7)
C10'	0.0582 (9)	0.0937 (12)	0.0684 (9)	0.0094 (8)	0.0059 (7)	0.0210 (8)
C11	0.0681 (10)	0.0721 (9)	0.0799 (10)	−0.0009 (8)	0.0014 (8)	0.0171 (8)
C11'	0.0741 (11)	0.0815 (11)	0.0865 (12)	0.0231 (9)	0.0164 (9)	0.0173 (9)
C12	0.0804 (11)	0.0594 (8)	0.0765 (10)	−0.0002 (8)	−0.0034 (8)	0.0037 (7)
C12'	0.0881 (13)	0.0732 (10)	0.0835 (11)	0.0182 (9)	0.0080 (9)	−0.0049 (9)
C13	0.0676 (9)	0.0636 (8)	0.0592 (8)	0.0094 (7)	0.0039 (7)	0.0005 (6)
C13'	0.0731 (11)	0.0717 (9)	0.0677 (9)	0.0098 (8)	−0.0004 (8)	−0.0062 (7)

### Geometric parameters (Å, º)

**Table:**

O1—C2	1.3763 (16)	C6—H6	0.9300
O1—C7A	1.3842 (19)	C6—C7	1.399 (3)
O1'—C2'	1.3791 (16)	C6'—H6'	0.9300
O1'—C7A'	1.3756 (17)	C6'—C7'	1.389 (2)
N2—H2A	0.916 (15)	C7—H7	0.9300
N2—H2B	0.919 (14)	C7—C7A	1.377 (2)
N2—C9	1.372 (2)	C7'—H7'	0.9300
N2'—H2'A	0.861 (15)	C7'—C7A'	1.374 (2)
N2'—H2'B	0.894 (14)	C8—C9	1.4129 (19)
N2'—C9'	1.373 (2)	C8—C13	1.404 (2)
N3—C2	1.2910 (18)	C8'—C9'	1.412 (2)
N3—C3A	1.4033 (19)	C8'—C13'	1.391 (2)
N3'—C2'	1.2889 (18)	C9—C10	1.403 (2)
N3'—C3A'	1.4001 (19)	C9'—C10'	1.402 (2)
C2—C8	1.455 (2)	C10—H10	0.9300
C2'—C8'	1.454 (2)	C10—C11	1.369 (3)
C4—H4	0.9300	C10'—H10'	0.9300
C4—C5	1.377 (3)	C10'—C11'	1.365 (3)
C4—C3A	1.385 (2)	C11—H11	0.9300
C4'—H4'	0.9300	C11—C12	1.382 (3)
C4'—C3A'	1.388 (2)	C11'—H11'	0.9300
C4'—C5'	1.379 (2)	C11'—C12'	1.374 (3)
C5—H5	0.9300	C12—H12	0.9300
C5—C6	1.384 (3)	C12—C13	1.363 (2)
C3A—C7A	1.369 (2)	C12'—H12'	0.9300
C3A'—C7A'	1.378 (2)	C12'—C13'	1.370 (2)
C5'—H5'	0.9300	C13—H13	0.9300
C5'—C6'	1.387 (3)	C13'—H13'	0.9300
			
C2—O1—C7A	103.41 (11)	C7A'—C7'—C6'	115.48 (16)
C7A'—O1'—C2'	103.83 (11)	C7A'—C7'—H7'	122.3
H2A—N2—H2B	118.9 (17)	C9—C8—C2	120.78 (13)
C9—N2—H2A	113.5 (12)	C13—C8—C2	120.11 (13)
C9—N2—H2B	117.1 (12)	C13—C8—C9	119.10 (14)
H2'A—N2'—H2'B	114.5 (19)	C3A—C7A—O1	108.32 (13)
C9'—N2'—H2'A	116.2 (14)	C3A—C7A—C7	124.26 (17)
C9'—N2'—H2'B	116.8 (13)	C7—C7A—O1	127.41 (16)
C2—N3—C3A	104.68 (12)	O1'—C7A'—C3A'	107.95 (13)
C2'—N3'—C3A'	104.70 (12)	C7'—C7A'—O1'	128.04 (14)
O1—C2—C8	116.10 (12)	C7'—C7A'—C3A'	124.01 (15)
N3—C2—O1	115.04 (13)	C9'—C8'—C2'	121.39 (13)
N3—C2—C8	128.86 (13)	C13'—C8'—C2'	119.30 (14)
O1'—C2'—C8'	115.96 (12)	C13'—C8'—C9'	119.31 (14)
N3'—C2'—O1'	114.88 (12)	N2—C9—C8	122.32 (14)
N3'—C2'—C8'	129.16 (13)	N2—C9—C10	120.15 (14)
C5—C4—H4	121.3	C10—C9—C8	117.49 (14)
C5—C4—C3A	117.38 (18)	N2'—C9'—C8'	122.22 (14)
C3A—C4—H4	121.3	N2'—C9'—C10'	120.26 (15)
C3A'—C4'—H4'	121.4	C10'—C9'—C8'	117.45 (15)
C5'—C4'—H4'	121.4	C9—C10—H10	119.1
C5'—C4'—C3A'	117.16 (16)	C11—C10—C9	121.80 (15)
C4—C5—H5	119.2	C11—C10—H10	119.1
C4—C5—C6	121.56 (18)	C9'—C10'—H10'	119.2
C6—C5—H5	119.2	C11'—C10'—C9'	121.53 (16)
C4—C3A—N3	131.24 (15)	C11'—C10'—H10'	119.2
C7A—C3A—N3	108.55 (13)	C10—C11—H11	119.7
C7A—C3A—C4	120.21 (15)	C10—C11—C12	120.57 (16)
C4'—C3A'—N3'	131.37 (14)	C12—C11—H11	119.7
C7A'—C3A'—N3'	108.64 (13)	C10'—C11'—H11'	119.5
C7A'—C3A'—C4'	120.00 (15)	C10'—C11'—C12'	120.94 (17)
C4'—C5'—H5'	119.1	C12'—C11'—H11'	119.5
C4'—C5'—C6'	121.84 (16)	C11—C12—H12	120.4
C6'—C5'—H5'	119.1	C13—C12—C11	119.20 (16)
C5—C6—H6	119.1	C13—C12—H12	120.4
C5—C6—C7	121.76 (17)	C11'—C12'—H12'	120.5
C7—C6—H6	119.1	C13'—C12'—C11'	118.99 (18)
C5'—C6'—H6'	119.2	C13'—C12'—H12'	120.5
C5'—C6'—C7'	121.52 (16)	C8—C13—H13	119.1
C7'—C6'—H6'	119.2	C12—C13—C8	121.83 (15)
C6—C7—H7	122.6	C12—C13—H13	119.1
C7A—C7—C6	114.83 (18)	C8'—C13'—H13'	119.1
C7A—C7—H7	122.6	C12'—C13'—C8'	121.77 (17)
C6'—C7'—H7'	122.3	C12'—C13'—H13'	119.1
			
O1—C2—C8—C9	178.67 (11)	C5—C4—C3A—N3	−178.83 (15)
O1—C2—C8—C13	−0.43 (18)	C5—C4—C3A—C7A	0.4 (2)
O1'—C2'—C8'—C9'	−179.01 (12)	C5—C6—C7—C7A	−0.2 (3)
O1'—C2'—C8'—C13'	0.9 (2)	C3A—N3—C2—O1	0.01 (15)
N2—C9—C10—C11	178.96 (15)	C3A—N3—C2—C8	179.91 (13)
N2'—C9'—C10'—C11'	−177.83 (16)	C3A—C4—C5—C6	−0.6 (3)
N3—C2—C8—C9	−1.2 (2)	C3A'—N3'—C2'—O1'	0.00 (16)
N3—C2—C8—C13	179.67 (14)	C3A'—N3'—C2'—C8'	−179.94 (13)
N3—C3A—C7A—O1	−0.04 (15)	C3A'—C4'—C5'—C6'	−0.1 (3)
N3—C3A—C7A—C7	179.31 (14)	C5'—C4'—C3A'—N3'	179.39 (15)
N3'—C2'—C8'—C9'	0.9 (2)	C5'—C4'—C3A'—C7A'	−0.4 (2)
N3'—C2'—C8'—C13'	−179.17 (15)	C5'—C6'—C7'—C7A'	−0.1 (3)
N3'—C3A'—C7A'—O1'	0.35 (16)	C6—C7—C7A—O1	179.19 (14)
N3'—C3A'—C7A'—C7'	−179.14 (14)	C6—C7—C7A—C3A	0.0 (2)
C2—O1—C7A—C3A	0.05 (14)	C6'—C7'—C7A'—O1'	−179.79 (15)
C2—O1—C7A—C7	−179.29 (15)	C6'—C7'—C7A'—C3A'	−0.4 (2)
C2—N3—C3A—C4	179.31 (15)	C8—C9—C10—C11	1.0 (2)
C2—N3—C3A—C7A	0.02 (15)	C7A—O1—C2—N3	−0.03 (15)
C2—C8—C9—N2	2.2 (2)	C7A—O1—C2—C8	−179.95 (11)
C2—C8—C9—C10	−179.83 (12)	C7A'—O1'—C2'—N3'	0.21 (15)
C2—C8—C13—C12	179.28 (14)	C7A'—O1'—C2'—C8'	−179.85 (12)
C2'—O1'—C7A'—C3A'	−0.33 (14)	C8'—C9'—C10'—C11'	−0.6 (2)
C2'—O1'—C7A'—C7'	179.14 (15)	C9—C8—C13—C12	0.2 (2)
C2'—N3'—C3A'—C4'	179.99 (15)	C9—C10—C11—C12	−0.7 (3)
C2'—N3'—C3A'—C7A'	−0.21 (16)	C9'—C8'—C13'—C12'	−0.4 (3)
C2'—C8'—C9'—N2'	−2.3 (2)	C9'—C10'—C11'—C12'	0.3 (3)
C2'—C8'—C9'—C10'	−179.43 (13)	C10—C11—C12—C13	0.1 (3)
C2'—C8'—C13'—C12'	179.70 (16)	C10'—C11'—C12'—C13'	0.0 (3)
C4—C5—C6—C7	0.5 (3)	C11—C12—C13—C8	0.2 (3)
C4—C3A—C7A—O1	−179.43 (13)	C11'—C12'—C13'—C8'	0.1 (3)
C4—C3A—C7A—C7	−0.1 (2)	C13—C8—C9—N2	−178.64 (13)
C4'—C3A'—C7A'—O1'	−179.83 (13)	C13—C8—C9—C10	−0.72 (19)
C4'—C3A'—C7A'—C7'	0.7 (2)	C13'—C8'—C9'—N2'	177.80 (15)
C4'—C5'—C6'—C7'	0.4 (3)	C13'—C8'—C9'—C10'	0.7 (2)

### Hydrogen-bond geometry (Å, º)

**Table:**

*D*—H···*A*	*D*—H	H···*A*	*D*···*A*	*D*—H···*A*
N2—H2*A*···N2′^i^	0.92 (2)	2.29 (2)	3.202 (2)	175 (2)
N2—H2*B*···N3	0.92 (1)	2.09 (2)	2.7679 (19)	129 (2)
N2′—H2′*A*···N2^ii^	0.86 (2)	2.52 (2)	3.359 (2)	164 (2)
N2′—H2′*B*···N3′	0.89 (1)	2.15 (2)	2.7913 (19)	129 (2)

Symmetry codes: (i) −*x*+1, *y*+1/2, −*z*+1/2; (ii) −*x*, *y*−1/2, −*z*+1/2.
